# Egr2 targets identify a population of dysfunctional T cells in the tumor microenvironment with immune modulatory properties

**DOI:** 10.1186/2051-1426-1-S1-P194

**Published:** 2013-11-07

**Authors:** Jason Williams, Yan Zheng, Thomas Gajewski

**Affiliations:** 1 Pathology, University of Chicago, Chicago, IL, USA; 2 Medicine, University of Chicago, Chicago, IL, USA

## 

The immune recognition of cancer provides potential for robust host control of tumors. Although the presence of tumor-infiltrating lymphocytes (TILs) indicates an endogenous anti-tumor response, immune regulatory pathways can subvert the effector phase and enable tumor escape. Negative regulatory pathways include anergy, expression of inhibitory receptors and ligands, metabolic dysregulation, and recruitment of suppressive cell populations. Recently, we have shown that the transcription factor Early Growth Response 2 (Egr2) is critical in controlling the anergic state by regulating the expression of DGK-α and -ζ. Gene expression profiling and Egr2 ChIP-Seq analysis revealed multiple Egr2-driven cell surface proteins in T cell anergy, including LAG-3, CRTAM, and 4-1BB. These data suggest that anergic cells may represent a differential functional state with potential immune regulatory capacity. In this study we used Egr2 targets as well as other previously defined molecules of T cell dysfunction, PD-1 and TIM-3, to characterize T cells in the context of the murine B16.SIY melanoma model. FACS and qPCR revealed subpopulations of CD8+ TILs expressing Egr2 in which PD-1 and LAG-3 are enriched. A major subset of these cells also expresses 4-1BB, TIM-3, and CRTAM. TCR repertoire analysis indicated TCRβ skewing in the marker-positive population suggesting oligoclonality, and arguing that this cell subset might represent the T cells with tumor specificity. Consistent with this notion, SIY-Kb pentamer staining revealed that the vast majority of CD8+ T cells specific for this model antigen expressed LAG-3, PD-1, and 4-1BB. In vitro stimulation of CD8+LAG-3+PD-1+ double positive (DP) population revealed blunted IL-2 transcription and proliferation compared to the double negative (DN) population. Despite the inability to make IL-2 and proliferate, these T cells produced high levels of IL-10, IFN-γ, CCL1, and CCL22, suggesting a potential immunoregulatory function. Preliminary data suggest that DP cells could suppress T cell proliferation in vitro and promote tumor growth in vivo. Our results suggest that the co-expression of LAG-3, PD-1, 4-1BB, and perhaps CRTAM may identify a critical subpopulation of dysfunctional TILs that are specific for tumor antigens and contribute to an immune suppressive tumor microenvironment. Ultimately, inhibiting or agonizing receptors on this subpopulation could have therapeutic relevance.

